# Non-Dispersive Extraction of Chromium(VI) by Cyphos IL102/Solvesso 100 Using the Pseudo-Emulsion-Based Strip Dispersion Membrane Operation

**DOI:** 10.3390/membranes14060129

**Published:** 2024-06-04

**Authors:** Francisco Jose Alguacil

**Affiliations:** Centro Nacional de Investigaciones Metalurgicas (CSIC), Avda, Gregorio del Amo 8, 28040 Madrid, Spain; fjalgua@cenim.csic.es

**Keywords:** non-dispersive extraction with strip dispersion, Cyphos IL102, chromium(VI), liquid membrane

## Abstract

The removal of chromium(VI) from an acidic (HCl) medium through non-dispersive extraction with strip dispersion (NDXSD) was investigated using a microporous PVDF membrane support in a permeation cell. The ionic liquid Cyphos IL102 (phosphonium salt) in Solvesso 100 was used as an organic phase. In NDXSD, the stripping phase (NaOH) is dispersed in the organic phase on the cell side with an impeller stirrer adequate to form a strip dispersion. This pseudo-emulsion phase (organic + strip solutions) provides a constant supply of the Cyphos IL102/Solvesso 100 to the membrane phase. Various hydrodynamic and chemical parameters, such as variation in the feed and pseudo-emulsion stirring speeds, HCl and Cr(VI) concentrations in the feed phase, and carrier concentration, were investigated. Results indicated that the best chromium(VI) transport was obtained under the following conditions: feed and pseudo-emulsion stirring speeds of 1000 min^−1^ and 600 min^−1^, respectively; an HCl concentration in the feed phase of 0.1 M; a chromium concentration of 0.01 g/L in the same phase; and carrier concentration in the organic phase in the 2–5–10% *v*/*v* range. From the experimental data, several mass transfer coefficients were estimated: a bulk diffusion coefficient of 3.1·10^−7^ cm^2^/s and a diffusion coefficient of 6.1·10^−8^ cm^2^/s in the membrane phase and mass transfer coefficients in the feed (5.7·10^−3^ cm/s) and membrane phases (2.9·10^−6^ cm/s). The performance of the present system against other ionic liquids and the presence of base metals in the feed phase were investigated.

## 1. Introduction

Ionic liquids (ILs) are a group of chemicals that are receiving considerable attention in research and application due to their recognition as green solvents. Due to their specific properties, they are considered alternatives to the use of some organic or inorganic chemicals and have the possibility of being used in customization (task-specific ILs) according to special applications, which expands their usefulness to various fields of interest, such as protein dissolution, stabilization, extraction, purification, etc. [1,2,3,4,5].

Chromium and its related compounds are widely used in different industries, resulting in the discharge of the element in different environments. This element (as with many others) is an essential micronutrient in humans and animals, but it is also a known carcinogenic (especially in the VI oxidation state) when its ingesta occurs at high concentrations; thus, its removal from generated effluents is of a general concern.

Thus, different technologies have been proposed to remove Cr(VI) from aqueous environments. Deep eutectic solvents formed by mixtures of tetra-n-octyl ammonium bromide and carboxylic acids [6] or amide C1_4_H_29_NO [7] are used to investigate the removal of this element from various aqueous media. Impregnating resins or polymer materials with DEHPA or ionic liquids (Cyphos IL 101, Cyphos IL 104 [8]) are technologies that have been investigated in the removal of chromium(VI), whereas adsorption using nanocomposites [9], biochar [10], or activated carbon [11] are recent examples of various adsorbents used in this role.

In the case of solvent extraction, some drawbacks related to this technology, especially the treatment of diluted metal solutions, lead to the development of alternatives to its use, with this being supported by liquid membrane (SLM) separation technology, which is of interest because it combines the kinetics and selectivity of solvent extraction and the simplicity of the membrane diffusion processes. SLMs belong to the advanced variation of extraction operation [12]. In conventional SLM technology (either in flat-sheet, spiral wound, or hollow fiber modules), the extraction and stripping processes are carried out simultaneously. In SLM separations, the feed and stripping or receiving aqueous solutions are separated by a hydrophobic membrane, which has been impregnated with the organic phase (normally comprised of the extractant or carrier and the diluent). This configuration facilitated the transport of the solute from the feed to the stripping phase. Of further interest is that this operational mode allowed the transport of a solute against its concentration gradient. However, some difficulties resulting from the stability or long-term performance of these SLMs led to the development of a more advanced SLM operational mode, such as the pseudo-emulsion-based strip dispersion, both in hollow fiber or flat-sheet operational modes [13,14,15,16,17]. Under this operation (see Section 2 for details), a pseudo-emulsion (organic and stripping phases) is formed by their mixing in a vessel, and due to that, the membrane support is hydrophobic, and the organic phase is immobilized into the membrane pores.

The present work investigates the removal of hazardous chromium(VI) from solutions using the ionic liquid Cyphos IlL02 dissolved in Solvesso 100 as the carrier phase. An advanced membrane operation such as non-dispersive extraction with strip dispersion is used to investigate such removal. In this membrane technology, the strip phase is dispersed into the organic phase to form a pseudo-emulsion phase, which disengages at the end of the operation to yield an organic phase and a strip solution containing the transported chromium(VI). Several hydrodynamic and chemical parameters influencing the transport of chromium(VI) are investigated, as well as the use of other ionic liquids and the presence of common base metals in the feed phase. From the strip phase, Cr(VI) was reduced to Cr(III) using hydrazine sulfate to render a less toxic solution with a certain degree of profitability (possible formation of pigments). Some mass transfer parameters are estimated from experimental results.

## 2. Materials and Methods

### 2.1. Materials

The ionic liquid Cyphos IL102 was obtained from Cytec Industries (Niagara Falls, ON, Canada) now Solvay (Paris, France), being its active group (trihexyl(tetradecyl)phosphonium bromide), and it was used without further purification. Cyphos IL101 (trihexyl(tetradecyl)phosphonium chloride) and Aliquat 336 (trioctyl(methyl)ammonium chloride) were obtained from Cytec Industries and Fluka (Switzerland), respectively. In the experimentation, the ionic liquids were dissolved in Solvesso 100 (99% aromatics, Exxon Chem, Iberia, Madrid, Spain). Dissolution of the ionic liquid is of interest due to the following factors: (I) It decreases the viscosity of the organic phase; this is of general importance in separation processes, i.e., solvent extraction, to facilitate phases disengagement, and in the case of membrane operations, it also facilitates the transport of a given solute, since the diffusion coefficient of the extractant–solute species in the membrane support is inversely proportional to the viscosity of the organic phase [18], (II) It gives an adequate range of extractant concentrations to a given system, and (III) in connection to (II), it avoids the use of unnecessary and, thus, unusable extractants in the global inventory of the system.

All the other chemicals in the present work were of AR grade.

The membrane support was Durapore GVHP4700 (Millipore, Burlington, MA, USA). This PVDF (poly(vinylidenefluoride) support has a diameter of 47 cm, with 0.22 μm nominal pore size, 125·10^−4^ cm thickness, and porosity of 75%.

### 2.2. Methods

**Figure 1:**
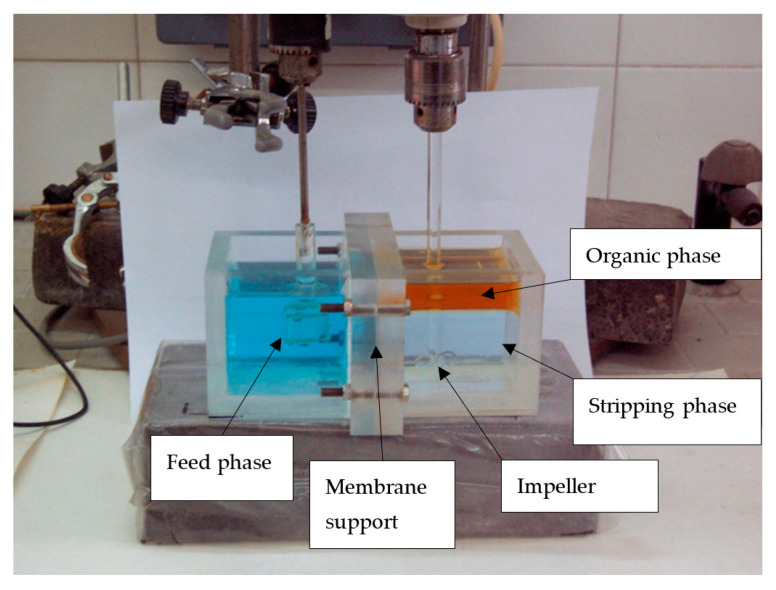
Non-dispersive extraction with strip dispersion. Location of the phases at t = 0.

The liquid membrane operation was performed with a flat-sheet membrane reactor that comprised two cells (200 mL each) provided with impeller stirrers with 2.5 cm diameter. The liquid membrane phase was prepared by soaking the membrane support overnight in a mixture of the ionic liquid and Solvesso 100 and leaving it to drip for twenty seconds before putting it in the cell. In the cell side corresponding to the organic and stripping phases, the impeller was located in the strip phase to provide dispersion of this phase into the continuous organic phase (Figure 1).

**Figure 2:**
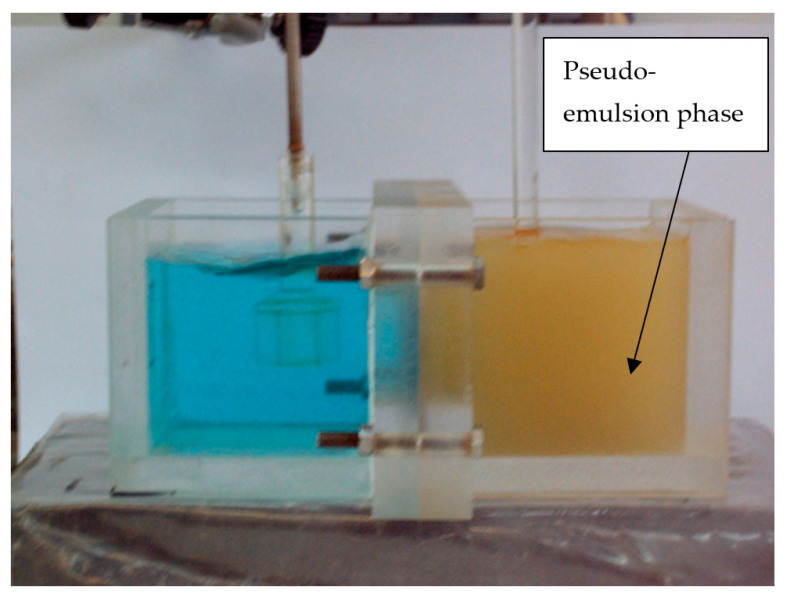
Non-dispersive extraction with strip dispersion. Experiment running at an elapsed time.

When the experiment ran, a pseudo-emulsion phase was immediately formed, and the metal was transported from the feed phase to the membrane phase and to the strip phase (Figure 2); mixing of the phases favored the stripping process.

**Figure 3:**
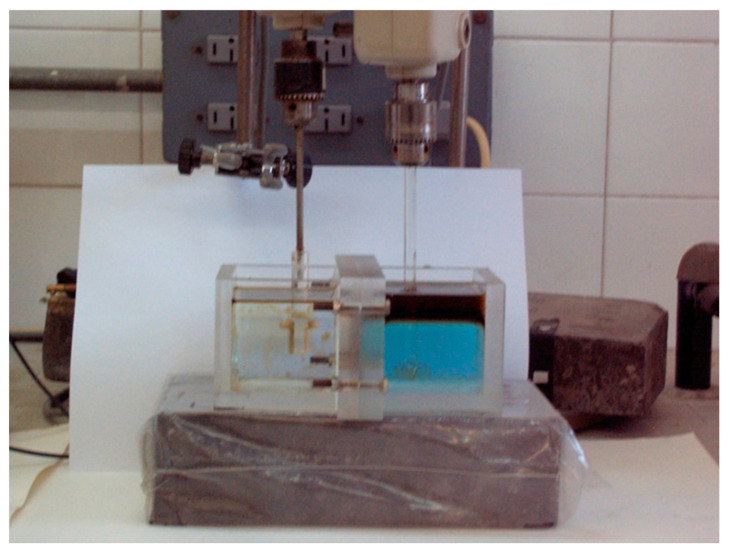
Non-dispersive extraction with strip dispersion. Phases at the end of the operation. The transport of the metal (blue color) from the feed to the strip phase can be observed.

At the end of the experiment, and after the stirring was stopped, the phases disengaged in a few minutes (Figure 3), and the system reached the initial disposition.

Membrane permeabilities were determined by monitoring chromium or metal concentration by atomic absorption spectrometry (Perkin Elmer 1100 B spectrophotometer, Seer Green, Great Britain) in the feed phase (or the stripping phase at the end of the experiment) as a function of time. The chromium analysis was found to be reproducible within ±2%. From the slope of the straight line obtained by plotting the left-hand side of the next equation versus time (t), the overall permeation coefficient (P) was estimated to be

(1) $$ln\frac{{\left[Cr\right]}_{f,t}}{{\left[Cr\right]}_{f,0}}=-\frac{AP}{V}\cdot t$$

where A is the effective membrane area (11.3 cm^2^), V is the volume (200 cm^3^) of the feed solution, and [Cr]_f,t_ and [Cr]_f,0_ are the chromium concentrations in the feed solution at an elapsed time t and time zero, respectively.

The percentage of chromium recovered in the strip phase was determined using the next relationship:

(2) $$\%R=\frac{{\left[Cr\right]}_{s,t}}{\frac{V_{f}}{V_{s}}({\left[Cr\right]}_{f,0}-{\left[Cr\right]}_{f,t}}\cdot 100$$

where [Cr]_s,t_ represents the metal concentration in the strip solution at an elapsed time, [Cr]_f,0_ and [Cr]_f,t_ have the same meaning as in Equation (1), and V_f_ and V_s_ are the volumes of the feed and stripping solutions, respectively.

## 3. Results and Discussion

**Figure 4:**
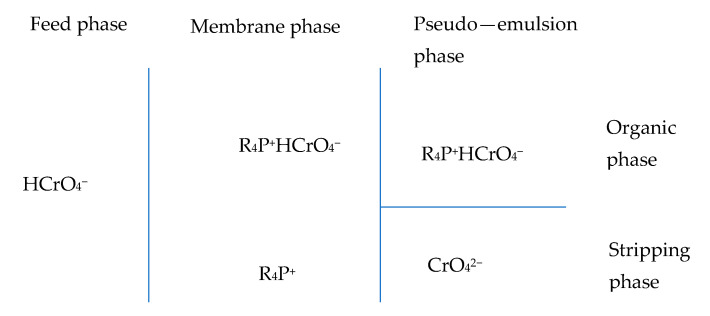
Distribution profile of the species involved in the facilitated transport process of chromium(VI) using Cyphos IL102 (R_4_P^+^ cationic moiety) as carrier.

Figure 4 shows a schematic profile of the species involved in the transport process once the experiment is stopped (Figure 3). The chemical reactions involved in the process were described elsewhere [19].

### 3.1. Influence of the Stirring Speed in the Feed and Pseudo-Emulsion Phases and Composition of the Stripping Phase on Cr(VI) Transport

**Figure 5:**
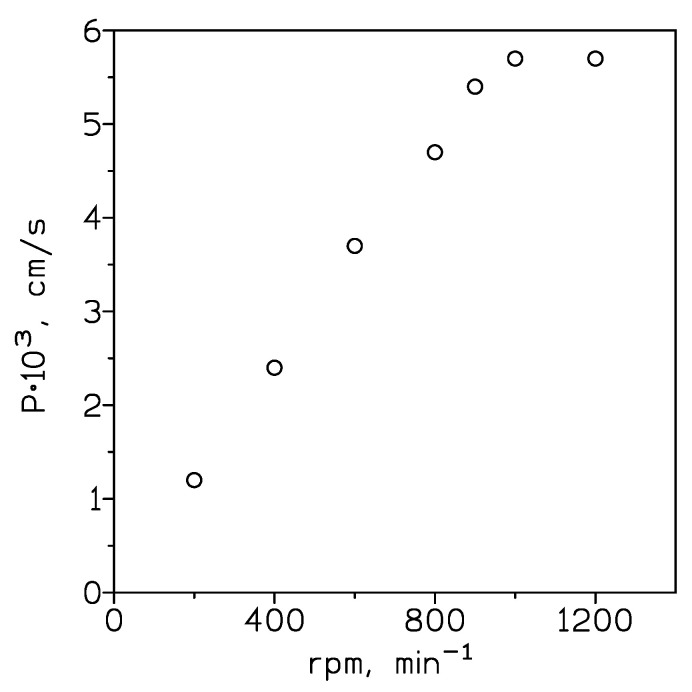
Variation of the permeation coefficient with the stirring speed of the feed phase. Feed phase: 200 cm^3^. Pseudo-emulsion phase (200 cm^3^): 100 cm^3^ organic phase + 100 cm^3^ stripping phase. Stirring speed pseudo-emulsion phase: 400 cm^3^. Temperature: 20 °C.

The influence of the variation of the stirring speed in the feed phase was investigated to optimize the uniform mixing of this solution and to minimize the thickness of the aqueous feed boundary layer with feed and pseudo-emulsion conditions maintained at 0.01 g/LCr(VI) in 0.1 M HCl and 10% *v*/*v* Cyphos IL102 in Solvesso 100 + 1 M NaOH, respectively. Results derived from these experiments are shown in Figure 5.

The permeation coefficient increased from 200 to 1000 min^−1^, indicating a progressive decrease in the boundary layer thickness, and then became independent of the stirring speed above 1000 min^−1^; thus, a minimum value of thickness was reached in this range of stirring speeds. The appearance of this plateau in the 1000–1200 min^−1^ region does not imply the complete elimination of the aqueous diffusion layer, but the minimization of the resistance due to it [20], resulting in a constant contribution of the diffusion of the chromium(VI) species to the mass transfer phenomena [21]. The stirring speed of 1000 min^−1^ in the feed phase was kept constant throughout the experimentation.

Using the same experimental conditions as above, the influence of the stirring speed in the pseudo-emulsion phase was also investigated. In this case, the speeds were varied in the 400–800 min^−1^ range, and the results indicated that this variation did not influence the metals transport. As a consequence, the stirring speed applied on the pseudo-emulsion phase was fixed at 600 min^−1^ in all the experiments.

It was described elsewhere [19] that NaOH solutions were effective in stripping Cr(VI) from loaded Cr(VI)-Cyphos IL102 organic phases. In the stripping process, Cr(VI) is released into the stripping solution as chromate species, regenerating the ionic liquid. Thus, within the present membrane methodology, NaOH solutions were also used for the stripping phases. The results of these experiments are summarized in Table 1. The permeation coefficients obtained using the different NaOH concentrations became almost independent of this variable; however, the recovery rate in the strip phase increases with the increase in the alkali concentration, though this variation was negligible in the 0.5–1 M NaOH concentrations range. It can also be observed that the chromium concentration in the stripping phase had an average concentration factor of 1.7 with respect to the initial chromium concentration in the feed phase. As a result of these experiments, 0.5 M NaOH was used as the stripping phase.

### 3.2. Influence of the HCl Concentration on Cr(VI) Transport

**Figure 6:**
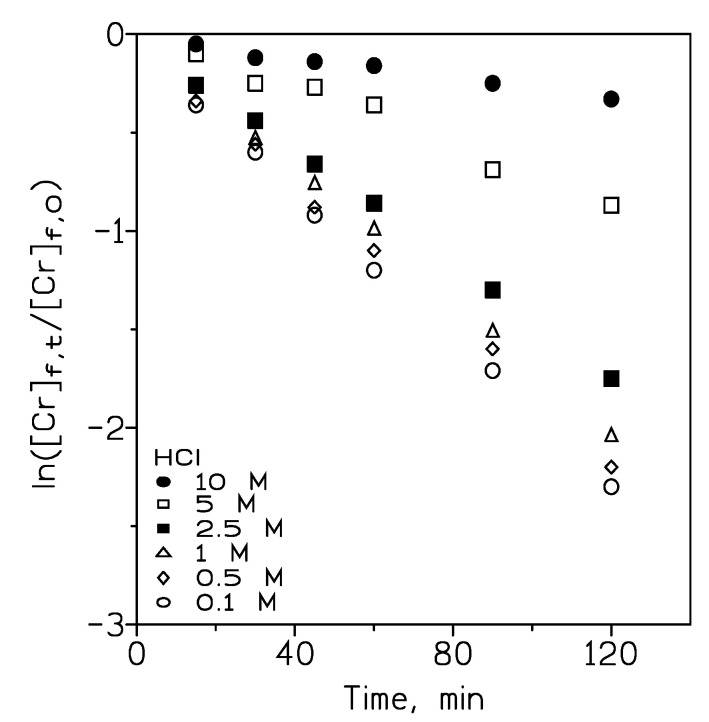
Chromium(VI) transport at various HCl concentrations in the aqueous phase. Temperature: 20 °C.

To assess the significance of the role of HCl concentration in the feed phase during the permeation of chromium(VI), HCl concentration variation investigations in the range 0.1–10 M were carried out in the presence of 0.01 g/l Cr(VI) in the feed phase (200 cm^3^), and in the presence of 10% *v*/*v* Cyphos IL102 in Solvesso 100 (100 cm^3^) + 0.5 M NaOH (100 cm^3^) as pseudo-emulsion phase (200 cm^3^). As seen from Figure 6 and Table 2, permeation of Cr(VI) decreased with the increase in the HCl concentration in the feed phase, and, thus, with the increase in the aqueous ionic strength, it negatively influenced the metal transport within the present system.

### 3.3. Influence of the Carrier Concentration in the Organic Phase on Cr(VI) Transport

In all liquid membrane technologies, the carrier or extractant plays a key role in making the transport operation efficient; in fact, the presence of this carrier facilitates the transport of the solute from the feed phase to the stripping phase by forming a specific solute–carrier complex, which also assisted the selectivity of the process. Moreover, a supported liquid membrane having no carrier immobilized within its pores results in a negligible solute transport. Thus, it is of the utmost importance to evaluate the influence of the Cyphos IL102 concentration in the organic phase on the transport of chromium(VI). The influence of the Cyphos IL102 concentration on the permeation of chromium(VI) was studied in the 0.6–10% *v*/*v* (0.01–0.17 M) concentration range.

**Figure 7:**
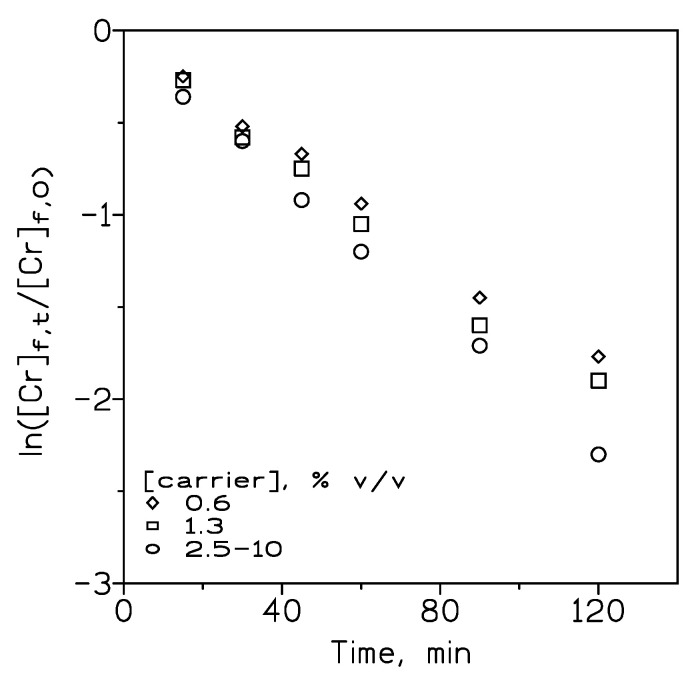
Chromium(VI) transport at various carrier concentrations in the organic phase. Feed phase (200 cm^3^): 0.01 g/L Cr(VI) in 0.1 M HCl. Pseudo-emulsion phase (200 cm^3^): 100 cm^3^ carrier in Solvesso 100 + 100 cm^3^ 0.5 M NaOH. Temperature: 20 °C.

As can be seen from Figure 7, the transport of Cr(VI) increases with the increase in the carrier concentration at 2.5% *v*/*v*, with no further increase up to 10% *v*/*v*. From these data, the permeation coefficients for chromium(VI) transport at the different carrier concentrations are given in Table 3.

These results show that the permeation coefficient reached a maximum or limiting value (5.7·10^−3^ M) at the carrier concentration of 2.5% *v*/*v* and levels off. This limiting value also represented the value of the mass transfer coefficient in the feed phase (∆_f_^−1^) and

(3) $$P_{lim}=\frac{D_{f}}{d_{f}}$$

where D_f_ is the metal diffusion coefficient in the feed phase (averaging 10^−5^ cm^2^/s [22], and d_f_ is the minimum thickness of the aqueous boundary layer. Thus, this d_f_ for the present system is estimated as 1.8·10^−3^ cm. The above results indicated that at low carrier concentrations in the membrane phase, diffusion of the Cr(VI)-Cyphos IL102 complex across the liquid membrane is the rate-determining step, whereas, in the 2.5–10% *v*/*v* concentration range, diffusion of the metal species across the aqueous boundary layer governed the transport process.

Assuming that the carrier concentration in the membrane phase is constant [23], the next equation allowed us to estimate the value of the diffusion coefficient of the chromium-carrier species in the membrane phase [18,24]:

(4) $$D_{m}=\frac{Jd_{m}}{\left[CyphosIL102\right]}$$

where d_m_ is the membrane thickness (125·10^−4^ cm), and J is the metal flux, which is calculated as

(5) $$J=P[Cr{]}_{f,0}$$

Thus, using a carrier concentration of 10% *v*/*v* (0.17 M), an initial chromium(VI) concentration in the feed phase of 0.01 g/L, and taking the correspondent value of the permeation coefficient (Table 3), the value of the flux is calculated as 1.1·10^−9^ mol/cm^2^s. Substituting this value in Equation (4), the value of D_m_ is estimated as 8.2·10^−8^ cm^2^/s.

### 3.4. Influence of the Chromium(VI) Concentration in the Feed Phase on Metal Transport

**Figure 8:**
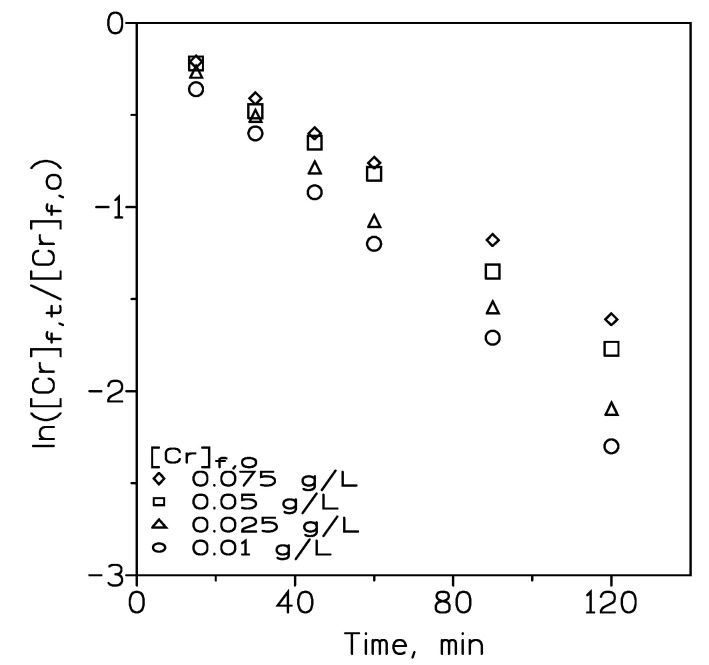
Chromium(VI) transport at various initial metal concentrations in the feed phase. Feed phase (200 cm^3^): Cr(VI) in 0.1 M HCl. Pseudo-emulsion phase (200 cm^3^): 100 cm^3^ 10% *v*/*v* Cyphos IL102 in Solvesso 100 + 100 cm^3^ 0.5 M NaOH. Temperature: 20 °C.

Figure 8 shows the variation in chromium(VI) transport with the variation of the initial metal concentration in the feed phase ranging from 0.01 to 0.075 g/L. Within this range of metal concentrations, the transport decreases with the increase in the metal concentration. Results in Figure 8 also indicated that an induction period was not observed, which makes Equation (19) valid for all the experiments.

Accordingly, as with the above, the permeation coefficients decreased with the increase in the chromium(VI) concentration in the feed phase (Table 4).

In this same table (Table 4), it can observed that the metal flux (J), calculated the same as in Equation (5), increases with the increase in the initial chromium(VI) concentration; thus, within the present experimental conditions, the transport of chromium(VI) is controlled by diffusion of metal species (HCrO_4_^−^, accordingly with the range of metal concentrations used in this work [19]). Metal recoveries in the stripping phase decreased with the increase in the initial metal concentration. After 2 h of reaction time, the metal concentrated in the stripping phase, except in the case of the feed solution containing 0.075 g/L Cr(VI), with this being probably attributed to the low recovery rate derived with the use of this metal concentration.

It is worth noting that chromium(VI) was transported against its concentration gradient; however, the time for these phenomena to occur is dependent on the initial metal concentration in the feed phase (Table 5). As it is seen, this period of time increased with the initial chromium(VI) concentration.

### 3.5. Diffusional Parameters and Contribution of Mass Transfer Resistances to the Overall Chromium(VI) Transport Process

As mentioned in Section 3.4., the mass transfer coefficient in the feed phase is estimated as 5.7·10^−3^ cm/s. The membrane mass transfer coefficient was estimated as [25]

(6) $$\;k_{m}=\frac{D_{m}\varepsilon }{\tau d_{m}}$$

where the tortuosity (τ) is 1.67, the porosity (ε) is 0.75, and the d_m_ is 125·10^−4^ cm. Thus, taking the value of 8.2·10^−8^ cm^2^/s for the membrane diffusion coefficient (see Equation (4)) for the chromium-carrier species in the organic solution, an estimated value of 2.9·10^−6^ cm/s is obtained for the present membrane system.

Furthermore, an effective membrane diffusion coefficient (D_eff,m_) of the chromium-ionic liquid species flowing across the membrane can be defined as [26]

(7) $$D_{eff,m}{=k_{m}d}_{m}\tau$$

and D_eff,m_ is estimated as 6.1·10^−8^ cm^2^/s, which is of the same magnitude order as the value of the membrane diffusion coefficient.

The diffusion coefficient of the chromium(VI) species in the bulk membrane phase (D_m,b_) can also be estimated as [27]

(8) $$D_{m,b}=\frac{D_{m}\tau^{2}}{\varepsilon }$$

Thus, the value of D_m,b_ is 3.1·10^−7^ cm^2^/s. The comparison of D_m_ and D_m,b_ values for the present system shows that the D_m_ value is lower than that of D_m,b_, which is attributable to the diffusional resistance caused by the membrane.

It was described in the literature [28] that the equilibrium and diffusional parameters involved in the transport process can be combined in an equation of the form

(9) $$\frac{1}{P}={∆}_{f}+\frac{{∆}_{m}}{C}$$

where C is a parameter involved in the extraction or transport process, which is considered the extraction equilibrium constant or constants and the concentration of the species that participated in the process. 1/P is the overall resistance, and Δ_f_ and Δ_m_ are the transport resistances in relation to diffusion by the feed boundary layer and the membrane, respectively.

In a transport process, the overall mass transfer resistance was the sum of the different resistances participating in the process, and thus, Equation (9) was rewritten as

(10) $$R=R_{f}+R_{m}$$

Table 6 shows the contribution (%R_f_^0^ and %R_m_^0^) of these various resistances to the overall resistance.

It was concluded that the diffusion by the aqueous feed boundary layer contributed to a major extent of the overall transport process. A mixed contribution of both aqueous and membrane diffusion under certain experimental conditions was also found.

### 3.6. Treatment of the Cr(VI)-Bearing Strip Phase

The recovery of chromium(VI) from the strip phase was investigated by treatment of this phase with solid hydrazine sulfate in order to reduce Cr(VI) to the Cr(III) oxidation state [29]. The redox reaction can be described as

(11) $$4CrO_{4}^{2-}+7H_{2}O+\frac{3}{2}N_{2}H_{6}^{2-}\to 4Cr(OH{)}_{4}^{-}+7OH^{-}+\frac{3}{2}N_{2}$$

This reduction is an instant reaction and allows for the obtaining of a potential pigment.

### 3.7. Comparison of the Performance of Cyphos IL102 against Other Ionic Liquids (Cyphos IL101 and Aliquat 336)

This investigation was performed using feed solutions of 0.01 g/L Cr(VI) in 0.1 M HCl and pseudo-emulsion phases containing 0.17 M of the single ionic liquid in Solvesso 100 + 0.5 M NaOH. The results of these experiments are shown in Table 7.

Results indicated that the performance of the three ionic liquids can be considered in similar terms since if the permeability coefficient is somewhat greater in the case of Aliquat 336, the recovery of chromium(VI) in the strip phase is better in the case of Cyphos IL102.

### 3.8. Transport of Chromium(VI) in the Presence of Base Metals

The transport of chromium(VI) was investigated in the presence of various base metals (Cu(II), Fe(III), Zn(II), and Co(II)). These experiments were carried out using binary solutions of Cr(VI) and each base metal at initial metal concentrations (each) of 1.9·10^−4^ M (roughly 0.01 g/L) in 0.1 M HCl. The pseudo-emulsion phase contained 10% *v*/*v* Cyphos Il102 in Solvesso 100 + 0.5 M NaOH. The results indicated that none of the investigated base metals permeate; thus, chromium(VI) can be selectively separated from these elements. However, it was found that the value of the chromium(VI) permeation coefficient decreased from 5.7·10^−3^ cm/s, using single Cr(VI) solutions, to an average value of 4.2·10^−3^ cm/s for the binary systems. This decrease can be attributable to the crowding or population effect due to the presence of these ions in the feed solution [30].

## 4. Conclusions

The transport of chromium(VI) from an HCl medium through a supported liquid membrane containing a Solvesso 100 solution of Cyphos IL102 was investigated. A microporous PVDF film, Durapore GVHP4700, was used as a solid support, whereas the pseudo-emulsion with strip dispersion membrane technology was used as advanced membrane operational mode. Chromium(VI) transport depends on a series of hydrodynamic and chemical variables. The aqueous boundary layer is minimized (d_aq_ = 1.8·10^−3^ cm) at stirring speeds in the 1000–1200 min^−1^ range, and carrier concentration is minimized in the membrane phase in the 2.5–10% *v*/*v* (4.3·10^−2^–1.7·10^−1^ M) range. An increase in the initial metal concentration in the feed phase produced a decrease in the permeation coefficient, but the metal flux increased, indicating an aqueous diffusion-controlled transport. The mass transfer coefficient in the aqueous film and the diffusivity of the Cr(VI)-Cyphos IL102 complex in the bulk organic membrane solution and in the membrane are also determined. The present system is comparable to the use of other ionic liquids, whereas with the use of Cyphos IL102/Solvesso 100 solution Cr(VI) is transported selectively from a series of base metals. From the strip solution and through the use of hydrazine sulfate, chromium(VI) was effectively reduced to the lesser toxic Cr(III) state, opening the possibility of obtaining a valuable pigment.

## Figures and Tables

**Table 1:** Influence of the stripping phase composition on Cr(VI) recovery in the strip phase.

Stripping Phase	ª % Cr(VI) Recovery	ª [Cr]_s,t_, g/L
0.1 M	90	0.016
0.5 M	96	0.017
1 M	98	0.018

ª After 2 h. Experimental conditions are the same as in Section 3.1.

**Table 2:** Chromium(VI) permeation coefficients at various HCl concentrations in the feed phase.

[HCl], M	P·10^3^, cm/s
0.1	5.7
0.5	5.5
1	5.0
2.5	4.7
5	2.1
10	0.83

Data are from Figure 6.

**Table 3:** Permeation coefficients for Cr(VI) at the different Cyphos IL102 concentrations in the organic phase.

[Cyphos IL 102], % *v*/*v*	P·10^3^, cm/s
0.6	4.6
1.3	5.0
2.5	5.7
5	5.7
10	5.7

Data are from Figure 7.

**Table 4:** Cr(VI) permeation coefficients, fluxes, and recoveries in the strip phase at various initial metal concentrations in the feed phase.

[Cr]_f,0_, g/L	P·10^3^, cm/s	J·10^9^, mol/cm^2^s	ª % Cr Recovery ([Cr]_s,t_, mg/L)	Concentration Factor
0.01	5.7	1.1	96 (17)	1.7
0.025	5.1	2.4	91 (40)	1.6
0.05	4.4	4.2	70 (58)	1.2
0.075	3.7	5.2	50 (60)	0.8

Data are from Figure 8. ª Time: 2 h.

**Table 5:** Estimated time from which Cr(VI) is transported against its concentration gradient.

[Cr]_f,0_, g/L	Time, min
0.01	22
0.025	24
0.05	33
0.075	51

Data are from Figure 8.

**Table 6:** Contribution of R_f_ and R_m_ to the overall chromium(VI) transport process.

Experimental Condition	R, s/cm	R_f_, min/cm	%R_f_^0^	%R_m_^0^
Stirring speed	175–833	175	100–21	0–79
HCl concentration	1204–175	175	15–100	85–0
Cr(VI) concentration	175–217	175	100–81	0–71
Carrier concentration	175–270	175	100–65	0–35

**Table 7:** Chromium(VI) transport using different ionic liquids.

Ionic Liquid	P·10^3^, cm/s	ª %Cr Recovery
Cyphos IL101	5.8	80
Cyphos IL102	5.7	96
Aliquat 336	6.0	77

ª Time: 2 h. Temperature: 20 °C.

## Data Availability

The original contributions presented in the study are included in the article, further inquiries can be directed to the corresponding author.
